# Association Between Physical Activity and Falls Among Iranian Older Adults: A Cross-Sectional Study from the Neyshabur Longitudinal Study on Ageing

**DOI:** 10.34172/jrhs.12716

**Published:** 2026-08-15

**Authors:** Zahra Azizabadi, Ensiyeh Mollanoroozy

**Affiliations:** ^1^Healthy Ageing Research Centre, Neyshabur University of Medical Sciences, Neyshabur, Iran; ^2^Noncommunicable Diseases Research Center, Neyshabur University of Medical Sciences, Neyshabur, Iran; ^3^Students Research Committee, Neyshabur University of Medical Sciences, Neyshabur, Iran; ^4^Workplace Health Research Center, Neyshabur University of Medical Sciences, Neyshabur, Iran

**Keywords:** Accidental falls, Aged, Exercise, Iran

## Abstract

**Introduction:**

Falls in the elderly are considered a major public health and social concern. In view of Iran’s growing ageing population, this study aimed to determine the prevalence of falls and to identify factors that influence them, especially the physical activity of older people.

**Study Design:**

A cross-sectional study.

**Methods:**

Data for this cross-sectional study were obtained from 7,462 participants in the Neyshabur Longitudinal Study of Ageing, registered in 2016-2018. Fall was the outcome variable. The effect of physical activity on falls was analyzed in conjunction with socioeconomic and medical history variables. Finally, statistical analysis was conducted using the chi-square test and logistic regression in SPSS 22.

**Results:**

The prevalence of falls was 20.4%. Moreover, adjusted logistic regression revealed that low physical activity (OR=1.19; 95% CI: 1.01–1.40), unemployment (OR=1.26; 95% CI: 1.04–1.54), and male gender (OR=s1.24; 95% CI: 1.02–1.5) were risk factors for falls (*P*<0.050).

**Conclusion:**

High physical activity may potentially play a beneficial role in reducing falls and their associated health outcomes, particularly in older individuals. Accordingly, any intervention to reduce falls should focus on physical activity, employment, and male gender.

## Introduction

 Aging is a multifactorial process that is characterized by physical, cognitive, and social changes. Globally, the share of the elderly population is projected to rise from 9.0–9.3% to about 16% by 2030, with Asia increasing from approximately 9.3% to 18.6% and Iran from 6.5% to 17.5% over the period 1995–2030.^1^ Age-related changes (e.g., declines in physical capacity, reduced economic opportunities, and limited social engagement) can contribute to loneliness and diminished quality of life, and may predispose individuals to comorbidities that complicate coping with daily challenges.^2^

 Declines in physical and mental performance in older adults occur at an average rate of about 1.5% per year, translating to roughly a 26% loss of functional capacity by age 70.^3^ Decreases in muscle strength, restricted joint range of motion, and diminished proprioception can impair balance, thereby increasing the risk of fall. Falls are a major public health concern, being a leading cause of unintentional injury-related mortality among older adults and a barrier to independence and well-being.^4,5^ Several interrelated factors, including muscle weakness, visual impairments, balance deficits, and unsafe environments, contribute to fall risk in this population.^6^ The World Health Organization reports that the prevalence of falls among older adults varies from 20% to 45% across different countries.^7^ In Asia, fall rates differ, with reported incidences ranging from 0.6% to 19.5%, influenced by ethnic, behavioral, and cultural factors.^8^ In Iran, available estimates place prevalence between 20% and 35%, with systematic reviews demonstrating around 30.9% among community-dwelling older adults and other studies indicating that about one in three elderly individuals experiences at least one fall.^7,9^

 Physical activity acts as a protective factor against non-communicable diseases (e.g., cardiovascular disease, stroke, and diabetes) and supports cognitive function, mood, and overall mental health. Regular activity enhances bone density, reduces adiposity, and lowers the risk of musculoskeletal injuries, thereby preserving functional capacity and promoting healthier aging.^10^

 Over the past two decades, interest in the physiological and psychological benefits of physical activity for the prevention of falls in older adults has increased due to the high prevalence and consequences of falls. Based on available data, approximately one-third of community-dwelling older adults fall each year, leading to injuries, loss of independence, and increased healthcare costs.^11,12^ Physical activity facilitates independent living; moreover, gait disorders elevate the risk of recurrent falls while reducing survival, underscoring the health relevance of activity level.^13-17^

 According to some systematic reviews, structured exercise programs can reduce fear of falling, improve self-efficacy, and promote adherence to activity, thereby lowering fall risk.^18^ Moreover, evidence suggests that programs emphasizing strength, balance, and coordination improve muscle performance and neuromuscular control, thus reducing fall risk.^18-20^ Given the modifiable nature of several fall-risk factors (e.g., lifestyle, education, employment, and living environment), this study examines whether the level of physical activity is associated with fall risk in the elderly. Accordingly, the present study aims to investigate the relationship between falls and physical activity level in this population.

## Methods

###  Study Population 

 The dataset originated from the Neyshabour Longitudinal Study on Aging (NeLSA), the aging arm of the Prospective Epidemiological Research Studies in Iran (PERSIAN). This multicenter initiative was implemented across four locations: Neyshabur in Razavi Khorasan Province (northeast Iran), Guilan (north), Tabriz (northwest), and Ardakan (central). For the present analysis, the focus was on the residents of Neyshabur aged 50–94 years who were enrolled between 2016 and 2018. Participants were identified through stratified random sampling of individuals registered with six health centers. In addition, eligibility required at least three years of continuous residence in Neyshabur, Iranian citizenship, and the absence of dementia, major depressive disorder, and disabling conditions that would preclude study participation. Of 9,220 individuals who met the inclusion criteria, 7,462, representing 4,831 households, provided written informed consent to participate, yielding a participation rate of 81%. Detailed descriptions of NeLSA sampling design and field implementation have been published previously.^21^

 For the present analysis, 7,275 eligible participants from NeLSA during baseline phase (2016–2018) were included, with complete data on physical activity assessment, fall history, and all required covariates. The inclusion criteria comprised age ≥ 50 years, participation in the baseline Phase (2016–2018) data collection, and complete information for physical activity and fall assessments. However, the exclusion criteria were incomplete physical activity data (n = 89), missing fall history (n = 0), and missing key covariates (n = 98). After applying these criteria, a total of 7,275 individuals remained for the final analysis.

###  Physical Activity 

 Information on physical activity was collected using the Physical Activity Scale for the Elderly through face-to-face interviews. This questionnaire consists of three sections. The first section focuses on leisure-time activities with six questions, and the second section is related to household activities with three questions. Likewise, the third section is associated with activities performed for pay or voluntarily and includes one question. Leisure-time activities (items 1–6) were rated on a 4-point Likert-type scale (1 = never, 2 = rarely, 3 = sometimes, 4 = often), and the remaining questions were scored as yes/no answers. Moreover, each activity was scored based on frequency (1 = less than one hour per day, 2 = between 1 and 2 hours per day, 3 = between 2 and 4 hours per day, 4 = more than 4 hours per day). Additionally, final scores were calculated by multiplying the weight of each activity by its frequency and ranged from 0 to 500, where higher scores indicated increased physical activity. Based on physical activity scores, individuals were classified into low-activity (0–66), moderate-activity (67–124), and high-activity (> 124) groups.

###  Fall Assessment

 Falls were assessed using a single question: “Have you experienced a fall in the past year?” (Yes/No). This question captured fall events that occurred in the 12 months preceding the interview. In this study, a fall was defined as “an unexpected event in which a person falls on the ground, floor, or lower level.” To identify those who had fallen, participants were asked: “Have you experienced a fall in the past year?” Those who answered “yes” were considered to have fallen, and this variable was coded dichotomously.

###  Other Variables

 Personal interviews were conducted by trained officers using a comprehensive questionnaire. Sociodemographic information included age, gender, marital status, education, and employment. In addition, lifestyle factors encompassed smoking status, body mass index, and medical history.

###  Statistical Analysis 

 Chi-square tests were used to compare the distribution of falls (yes/no) across different categories of sociodemographic characteristics and health-related variables. Moreover, the relationship between individual factors and outcome measure (falls) was assessed using crude logistic regression. Further, independent associations of risk factors were examined through adjusted logistic regression. The results from regression models are presented as odds ratios (ORs) and 95% confidence intervals (CIs). All reported *P*-values were based on two-sided tests, where the level of statistical significance was set at *P* < 0.05. To control familywise error arising from simultaneous evaluation of 10 prespecified predictors, a conservative Bonferroni correction was implemented, setting the two-sided significance criterion to α_adj = 0.05/10 = 0.005. All statistical analyses were conducted using SPSS software, version 22 (SPSS Inc., Chicago IL, USA).

## Results

 Data from 7,462 participants were employed in the current analysis. The mean ± standard deviation of the age of participants was 61.0 ± 8.14 years, and the majority of them had primary education. Furthermore, most participants (90%) lived with another person. The prevalence of overweight and obesity was 43.0% and 29.9% respectively, while 10.9% of participants were reported being smokers. More detailed characteristics of the participants are provided in Table 1.

**Table 1:** Distribution of Fall by Demographic and Prior Medical History, Neyshabur, Iran (2016-2018)

**Variables**	**Study Population (N=7462)**		**Fall**		
	**Frequency**	**%**	**Frequency**	**%**	***P*** **-Value**
Age group (year)					
50-59 60-69 70-79 ≥ 80	4,010	53.7	779	51.1	0.008
	2,338	31.3	482	31.6	
	877	11.8	197	12.9	
	237	3.2	65	4.3	
Gender					
Female Male	3,962	53.1	877	57.6	0.001
	3,500	46.9	646	42.4	
Education					
Illiterate Primary Secondary Diploma University	1,934	26.3	339	26.4	0.172
	2,514	34.1	501	33.1	
	666	9.0	158	10.4	
	1,527	20.7	319	21.1	
	723	9.8	135	8.9	
Body mass index (kg/m^2^)					
Underweight Normal Overweight Obese	130	1.8	21	1.4	0.158
	1,822	25.2	348	23.5	
	3,105	43.0	647	43.7	
	2,160	29.9	464	31.4	
Smoking					
Yes No	760	10.9	159	10.9	0.521
	6,189	89.1	1,295	89.1	
Occupation status					
Employed Unemployed	3,494	47.5	642	16.0	0.001
	3,877	52.5	869	22.4	
Hypertension					
Yes No	2,894	43.1	621	45.0	0.052
	3,825	56.9	757	54.9	
Diabetes					
Yes No	1,937	26.3	429	28.4	0.016
	5,435	73.7	1,077	71.6	
Living alone					
Yes No	748	10.1	172	11.4	0.039
	6,636	89.9	1,337	88.6	
Physical activity scale for the elderly					
Low activity Moderate activity High activity	3,861	53.0	780	20.2	0.011
	1,306	18.0	306	23.0	
	2,108	29.0	406	19.2	

 The prevalence of falls in the total population was 20.4%. Similarly, the distribution of physical activity levels among participants was as 53% (low), 18% (moderate), and 29% (high). More information on the distribution of this disorder across independent variables is presented in Table 1. Based on the results, falls were less frequent in older age groups (*P* = 0.008). In addition, age, gender, occupational status, diabetes, living alone, and physical activity were observed to significantly influence falling (*P* < 0.039). Conversely, no differences in fall were noted concerning body mass index, education, smoking, or hypertension (Table 1).

 The frequency distribution of falls according to physical activity levels is shown in Table 1. Among participants, 20.2%, 23.04%, and 19.2% of those with low, moderate, and high physical activities, respectively, reported at least one history of falling during the past year. There was a significant difference across physical activity levels (low, moderate, and high).

 Logistic regression analysis was performed to explore both crude and adjusted associations between physical activity levels and falls while considering other health-related predictors (Table 2). In both crude and adjusted logistic regression models assessing physical activities and other predictors in relation to falls, several estimates suggested possible trends, but no association crossed the Bonferroni-adjusted threshold of 0.005. It should be noted that these results are now reported with uncorrected–Bonferroni significance levels for transparency. No significant association was found in the crude models; however, there was a significant association between physical activity levels and falls in adjusted models.

**Table 2:** Binary Logistic Regression Analysis of Fall in the Elderly in Neyshabur, Iran (2016-2018)

**Variables**	**Crude OR (95% CI)**	***P*** **-Value**	**Adjusted OR (95% CI)**	***P*** **-Value**
Physical activity scale for the elderly				
Low activity Moderate activity High activity	1.00 (0.88, 1.14)	0.922	1.19 (1.01, 1.40)	0.029
	0.92 (0.77, 1.09)	0.350	0.90 (0.75, 1.09)	0.299
	Ref.		Ref.	
Age group (year)				
50-59 60-69 70-79 ≥ 80	Ref.		Ref.	
	0.92 (0.81, 1.05)	0.252	0.87 (0.75, 1.01)	0.072
	0.83 (0.69, 0.99)	0.042	0.73 (0.59, 0.91)	0.006
	0.63 (0.47, 0.85)	0.003	0.46 (0.32, 0.67)	0.005
Gender				
Female Male	Ref.		Ref.	
	1.25 (1.12, 1.40)	0.000	1.24 (1.02, 1.5)	0.025
Hypertension				
No Yes	Ref.		Ref.	
	0.90 (0.80, 1.01)	0.097	0.96 (0.84, 1.09)	0.553
Living alone				
No Yes	Ref.		Ref.	
	1.18 (0.98, 1.41)	0.067	1.00 (0.80, 1.24)	0.985
Diabetes				
No Yes	Ref.		Ref.	
	0.86 (0.76, 0.98)	0.029	0.89 (0.77, 1.03)	0.142
Education				
Illiterate Primary Secondary Diploma University	0.88 (0.71, 1.09)	0.262	1.11 (0.80, 1.24)	0.446
	0.92 (0.74, 1.13)	0.454	1.01 (0.79, 1.30)	0.884
	0.73 (0.57, 0.95)	0.021	0.79 (0.59, 1.06)	0.120
	0.86 (0.69, 1.08)	0.221	0.88 (0.68, 1.13)	0.337
	Ref.		Ref.	
Body mass index (kg/m^2^)				
Underweight Normal Overweight Obese	Ref.		Ref.	
	0.81 (0.50, 1.32)	0.408	0.67 (0.38,1.19)	0.180
	0.73 (0.45, 1.17)	0.198	0.61 (0.35, 1.08)	0.094
	0.70 (0.43, 1.13)	0.151	0.64 (0.36, 1.13)	0.130
Occupation status				
Employed Unemployed	Ref.		Ref.	
	1.28 (1.14, 1.43)	0.000	1.26 (1.04, 1.54)	0.016
Smoking				
No Yes	Ref.		Ref.	
	1.00 (0.83, 1.20)	0.998	0.80 (0.64, 0.99)	0.053

*Note*. CI: Confidence interval; OR: Odds ratio

 In univariate analysis, male gender (OR = 1.25; 95% CI: 1.12–1.40; *P* < 0.001) and unemployment (OR = 1.28; 95% CI: 1.14–1.43; *P*< 0.001) were associated with an increased risk of falls. By contrast, being 70–80 years old (OR = 0.83; 95% CI: 0.69–0.99; *P*= 0.042), being over 80 years old (OR = 0.63; 95% CI: 0.47–0.85; *P*= 0.003), having diabetes (OR = 0.86; 95% CI: 0.76–0.98; *P*= 0.029), and achieving secondary education (OR = 0.73; 95% CI: 0.57–0.95; *P*= 0.021) were negatively related to falls.

 In the adjusted model, low physical activity emerged as a modest predictor for an increased risk of fall among the elderly population. Specifically, the risk of falls in elderly with low physical activity was 1.19 times higher than those with high physical activity (OR = 1.19; 95% CI: 1.01–1.40; *P*= 0.029). Finally, male gender (OR = 1.24; 95% CI: 1.02–1.50; *P*= 0.025), and unemployment (OR = 1.26; 95% CI: 1.04–1.54; *P*= 0.016) showed a significant positive association with falls.

## Discussion

 This cross-sectional analysis of Iranian older adults from the NeLSA cohort examined the association between physical activity and falls. Our main findings indicated that low physical activity is significantly associated with the increased odds of falls, even after controlling for sociodemographic and health-related factors. Additionally, male gender and unemployment were serious risk factors for falls in this population. Based on our results, more than 20% of participants had a history of falling in the past year. Consistent with our findings, Iranian adults are reported to have approximately equal fall rates,^22^ with similar trends observed in other Middle Eastern countries.^23^ Previous research highlighted a wide range of determinants for falls, with primary factors encompassing physiological declines,^24^ cognitive impairments,^25^ chronic health conditions,^26,27^ medications,^24,26^ and sensory impairments.^25^ While some observational studies suggested a relationship between physical activity and falls,^28,29^ the impact of confounding factors (e.g., occupation status, education, lifestyle choices, and overall health status) on these associations has not been thoroughly explored yet.

 Low physical activity is indeed an important predictor of falls in the elderly, as evidenced by multiple studies.^28,29^ The relationship between low physical activity and increased fall risk is compounded by other factors, such as fear of falling and depression. These factors frequently interact synergistically, exacerbating the risk of falls among older adults. Some studies highlight the importance of maintaining an active lifestyle to mitigate fall risk and emphasize the need for targeted interventions for those with low physical activity levels.^30,31^

 A study involving older Taiwanese community-dwellers found that low physical activity, when combined with fear of falling, significantly increased the risk of falls. The adjusted odds ratio for fall risk was 2.33, indicating more than a twofold increase in risk for those with both factors compared to those with neither.^32^ Similarly, research conducted in Brazil demonstrated that elderly individuals with lower levels of physical activity exhibited poorer functional mobility, as measured by the Timed Up and Go test. In addition, participants classified as inactive took longer to complete the Timed Up and Go test, implying a higher fall risk.^33^

 The consensus among many studies suggests that physical exercise, particularly strength and balance training, can improve various factors of physical performance in older adults, including balance, lower extremity strength, and mobility. This improvement can lead to a decrease in fall-related injuries^34^ and is generally recommended by some organizations, such as the World Health Organization and the U.S. Preventive Services Task Force.^35^ Despite the positive outcomes associated with exercise programs, several key studies show minimal to no significant reduction in fall rates. For instance, a randomized controlled trial involving elderly residents in long-term care found no difference in fall rates between those participating in structured exercise and those in a control group engaged in chair-based activities.^36^ Additionally, a review by the Cochrane Database reflected heterogeneous findings across various studies, with some interventions yielding statistically insignificant results.^37^ Older adults frequently demonstrate different health and personal factors, such as cognitive impairment, vision issues, and concurrent health conditions, which can complicate the outcomes of exercise interventions.^38^ Consequently, although some studies reported a clear association between physical activity and reduced fall risk, other studies revealed no protective effects.^39,40^

 According to our results, the relationship between unemployment and increased risks of falls in older adults is underscored by several studies, highlighting how economic instability and job loss can significantly impact the physical health and safety of this population. Based on evidence, older adults experiencing involuntary job loss, especially those approaching retirement, face various health challenges, including the increased risks of functional disability. A study using data from the Health and Retirement Study demonstrated that these negative impacts are particularly prevalent in women and those with lower socioeconomic status.^41^ It should be noted that the stress associated with job loss leads to adverse health outcomes (e.g., increased anxiety, social withdrawal, and harmful health behaviors), probably contributing to a heightened risk of falls due to reduced physical activity and deterioration of mental health.^26^

 Studies emphasize a relationship between socioeconomic status and psychological factors (e.g., fear of falling and perceived risk), which can discourage older adults from engaging in preventative behaviors, such as physical exercises. This perception may be more prevalent among those with a history of job loss or economic hardship.^42^ In contrast to our findings, those of some studies revealed that working seniors might actually report lower fall rates compared to their unemployed peers.^26,43^

 Gender plays a significant role in predicting fall risk among the elderly, with distinct patterns observed between men and women. Furthermore, perception of fall risks varies by gender. In discussions about falls, men often present an analytical approach, focusing on risk assessment and behavior modification, while women may engage in controlling extrinsic factors in order to mitigate risk. This aligns with the finding that men generally have a more relaxed attitude toward physical limitations, which may potentially lead to riskier behaviors and consequently a higher likelihood of fall risk.^44^ Beyond biological differences, environmental contexts further contribute to gender disparities in fall risks. For instance, men are more likely to engage in higher levels of physical activity and risky behavior, which are correlated with increased fall incidents.^45^ Additionally, this represents a crucial area for preventive strategies in clinical settings where male patients may benefit from more tailored fall prevention protocols. In examining frailty as a risk factor for falls, research has shown that older men with frailty demonstrated a higher risk of falling than older women. Specifically, a recent systematic review reported that the relative risk of falls in older men with frailty was 1.94 compared to 1.44 for women.^45^ The findings of some studies, contrary to our results, confirmed that women are more prone to falls compared to men, due to a combination of physiological, psychological, and environmental factors.^44,46,47^

 Regarding other risk factors for falls, such as smoking,^48^ hypertension,^45,49^ obesity, education and diabetes,^50,51^ there were discrepancies between our findings and those of some previous studies. Our results represented no significant association between some predictors of falls, including smoking, hypertension, diabetes, obesity, and education with an increased risk of falls. The variations in findings across studies could be attributed to genetic factors, lifestyle choices, dietary habits, and environmental conditions, or the specific age ranges of participants, as our study focused on the elderly population. Further, methodological and contextual considerations may account for the null associations. Furthermore, the cross-sectional design precludes the assessment of temporal dynamics, cumulative disease burden, and treatment trajectories. Likewise, differences in the prevalence, severity, and clinical control of these conditions, such as superior medication adherence and tighter control of diabetes or hypertension, could attenuate observable relationships in our cohort relative to other populations. Moreover, cultural and environmental features specific to the Iranian context (e.g., housing design, social support structures, and healthcare access) may modify risk pathways and dilute direct associations with medical diagnoses. ^48,51^

 It is important to acknowledge the limitations of this study. First, the number of key determinants included in the statistical model may have been insufficient, although efforts were made to incorporate the most relevant variables with complete data. Likewise, the data analyzed in this study were drawn from the early years of the cohort study; therefore, future follow-ups may provide additional insights and clarify the role of these risk factors over time. Ultimately, it should be acknowledged that we did not collect data on the number of falls per participant.

**Highlights**

- The prevalence of falls in the total population was 20.4%.
- Low physical activity was moderately associated with the increased odds of falls in older adults.
- Male gender and unemployment were identified as significant risk factors for falls.
- Moderate-to-high physical activity levels may serve as protective factors against falls in the elderly population.

## Conclusion

 Our study found a statistically significant but modest association between low physical activity and falls in Iranian older adults. This finding should be cautiously interpreted and confirmed in larger longitudinal studies. Nevertheless, given that physical activity is a modifiable risk factor with multiple health benefits beyond fall prevention, promoting physical activity remains a valuable component of comprehensive fall prevention programs for older adults. Moreover, male gender, and being unemployed were identified as risk factors for falls. Accordingly, future interventions should adopt multifactorial approaches that address physical activity alongside other established risk factors.

## Acknowledgments

 This study was supported by Neyshabur University of Medical Sciences (project No. 14030518). We further express our gratitude and appreciation to PERSIAN Central Committee, Vice-Chancellor in Research of Neyshabur University of Medical Sciences, Research Department of Neyshabur University of Medical Sciences, all staff of Neyshabur Longitudinal Study on Ageing, and all participants who were involved in this project.

## Artificial Intelligence Use Disclosure

 In this study, no AI tools were used to directly generate or analyze data, interpret results, or draft any sections of this manuscript. All data collection, analyses, and conclusions are the sole work of the authors. Although AI tools may have been consulted for non-substantive tasks (e.g., language editing or translation), they did not contribute to the content or interpretation of the study.

## Competing Interests

 The authors have no competing interests associated with the material presented in this study.

## Ethical Approval

 NeLSA was approved by the Ethics Committee of Neyshabur University of Medical Sciences (record No. IR.NUMS.REC, 1394.35), and the PERSIAN Cohort Study received ethical approval from Tehran University of Medical Sciences (record No. IR.TUMS.DDRI.REC.1396.1). In addition, written informed consent was obtained from all participants, and they were free to withdraw from the study at any time and for any reason without any consequences. Moreover, all methods were performed in accordance with the relevant guidelines and regulations, and all procedures were in accordance with the Helsinki Declaration. The current study also received an ethical approval from Neyshabur University of Medical Sciences (ethical No. IR.NUMS.REC.1404.030).

## Funding

 This study was financially supported by Neyshabur University of Medical Sciences (Project No.14030518).
